# Methylomic and phenotypic analysis of the ModH5 phasevarion of *Helicobacter pylori*

**DOI:** 10.1038/s41598-017-15721-x

**Published:** 2017-11-23

**Authors:** Yogitha N. Srikhanta, Rebecca J. Gorrell, Peter M. Power, Kirill Tsyganov, Matthew Boitano, Tyson A. Clark, Jonas Korlach, Elizabeth L. Hartland, Michael P. Jennings, Terry Kwok

**Affiliations:** 1 Department of Microbiology, Monash University, Clayton, 3800 Victoria Australia; 2 Department of Microbiology and Immunology, University of Melbourne at the Peter Doherty Institute for Infection and Immunity, Parkville, 3010 Victoria Australia; 3 Infection and Immunity Program, Biomedicine Discovery Institute, Monash University, Clayton, 3800 Victoria Australia; 4 Department of Biochemistry and Molecular Biology, Monash University, Clayton, 3800 Victoria Australia; 5 Cancer Program, Biomedicine Discovery Institute, Monash University, Clayton, 3800 Victoria Australia; 6 Institute for Glycomics, Griffith University, Gold Coast, Queensland 4222 Australia; 7 Bioinformatics Platform, Monash University, Clayton, 3800 Victoria Australia; 8 Pacific Biosciences, Menlo Park, CA 94025 USA; 9 Present Address: Department of Molecular and Translational Science, Hudson Institute of Medical Research, Monash University, Clayton, 3800 Victoria Australia

## Abstract

The *Helicobacter pylori* phase variable gene *modH*, typified by gene HP1522 in strain 26695, encodes a N^6^-adenosine type III DNA methyltransferase. Our previous studies identified multiple strain-specific *modH* variants (*modH1* – *modH19*) and showed that phase variation of *modH5* in *H. pylori* P12 influenced expression of motility-associated genes and outer membrane protein gene *hopG*. However, the ModH5 DNA recognition motif and the mechanism by which ModH5 controls gene expression were unknown. Here, using comparative single molecule real-time sequencing, we identify the DNA site methylated by ModH5 as 5′-G^m6^ACC-3′. This motif is vastly underrepresented in *H. pylori* genomes, but overrepresented in a number of virulence genes, including motility-associated genes, and outer membrane protein genes. Motility and the number of flagella of *H. pylori* P12 wild-type were significantly higher than that of isogenic *modH5* OFF or Δ*modH5* mutants, indicating that phase variable switching of *modH5* expression plays a role in regulating *H. pylori* motility phenotypes. Using the flagellin A (*flaA*) gene as a model, we show that ModH5 modulates *flaA* promoter activity in a GACC methylation-dependent manner. These findings provide novel insights into the role of ModH5 in gene regulation and how it mediates epigenetic regulation of *H. pylori* motility.

## Introduction

*Helicobacter pylori* is a highly prevalent human pathogen infecting >50% of the population worldwide^1,2^ and is a causative factor of chronic gastritis, peptic ulcer and gastric cancer^3,4^. *H. pylori* is also characterized by its remarkable ability to persistently colonize its host. The need for *H. pylori* to adapt to the hostile, highly variable human gastric environment whilst having to subvert and evade host immune responses underpins its marked inter-strain genetic diversity. Phase variation, the high-frequency reversible on/off switching of gene expression^5^, is one of the major mechanisms employed by *H. pylori* to achieve genetic variation. Phase variable genes typically contain simple tandem DNA repeats within the open reading frame or promoter region. The reversible loss or gain of these repeats, mediated by slipped strand mispairing, can alter promoter specificity or cause frame-shift mutations that lead to inactivation or truncation of the resultant gene product. We previously reported that several clinically important bacterial pathogens, including *H. pylori*, express phase variable N^6^-adenosine type III DNA methyltransferases (Mod) that contribute to global changes in gene expression^6–11^. The target genes regulated by these phase variable DNA methyltransferases are collectively referred to as phase variable regulons, or phasevarions. Phasevarion-associated DNA methyltranserases are emerging as important epigenetic regulators of prokaryotic virulence factor and surface antigen expression^12–14^.

One such phase variable type III DNA methyltransferase of *H. pylori* is encoded in the prototypical genome strains J99 and 26695 by *jhp1411* and *hp1522*, respectively; we refer to these, and the homotypic loci from all *H. pylori* strains, as *modH* ^11^. Phase variable regulation of *modH* is mediated by random alteration in the length of a guanosine (poly-G) repeat tract located 104 bp downstream of the gene region encoding the ModH DNA recognition domain (DRD). Poly-G tract length variation results in translation of either a full-length functional ModH enzyme (“ON”), or a truncated, inactive protein (“OFF”). The *modH* gene shows interstrain sequence diversity of the DRD region with 19 allelic types now identified (types *modH1* – *modH19*)^11,15^. The prototypical strains J99 and 26695 carry alleles *modH1* (*jhp1411*) and *modH2* (*hp1522*), respectively, with the latter recently found to be inactive in 26695^16^. The first *H. pylori* ModH phasevarion to be examined was *modH5* (*hpp12_1497*), carried by *H. pylori* strain P12^11^. In that study, *modH5* was identified as one of the most prevalent among the 17 *modH* alleles identified at the time, and was carried by ~15% of isolates examined. ON-phase of P12 *modH5* was shown to positively regulate the expression of motility-associated genes *flaA*, *flgE* and *hpp12_0904*, and negatively regulate the expression of motility-associated gene *hpp12_0255* and outer membrane colonization factor gene *hopG* ^17,18^. More recently, the ModH1 phasevarion of *H. pylori* strain J99 has been examined and shown to also contain several motility-associated genes, including *flgE* ^19^. However, in contrast to the ModH5 phasevarion, *flgE* is positively regulated by the ModH1 OFF-phase. In line with these observations of ModH-mediated gene regulation, a role for ModH phase switching in *H. pylori* colonization has been implicated by the findings of a recent mouse model study^20^. Despite the predictive transcriptional evidence, the phenotypic role of ModH phase switching in *H. pylori* motility had not been examined. Furthermore, the DNA target recognition motif of ModH5 and the molecular mechanism by which ModH5 mediates epigenetic regulation of gene expression remained unknown.

The development of Single Molecule Real-Time (SMRT) sequencing enables both genomic and epigenomic information to be derived from bacterial DNA^21,22^ and has been used to characterize the methylomes of several pathogenic bacteria including *Escherichia coli* ^21^, *Moraxella catarrhalis* ^7^, *H. pylori* ^16,19,23,24^, *Campylobacter coli* ^25^, *Neisseria menigitidis* ^26^, non-typeable *Haemophilus influenzae* ^6^ and *Mycobacterium tuberculosis* complex species^27^. In particular, the data obtained have provided important insights into the activities and target DNA recognition motifs of numerous bacterial DNA methyltransferases. Here we report on identification of the target DNA recognition motif of *H. pylori* ModH5 methyltransferase using SMRT sequencing. This, together with findings from phenotypic and promoter analyses, provided novel insight into the mechanism by which ModH5 epigenetically controls the expression of virulence genes important for *H. pylori* colonization and survival.

## Results

### SMRT methylome characterization of *H. pylori* strain P12 identifies 5′-G^m6^ACC-3′ as the methylation target site of ModH5

Extensive genetic diversity of the *modH* DNA recognition domain strongly suggests that different ModH allelic variants may methylate different target DNA motifs. To identify the ModH5 methylation target site, we performed SMRT sequencing of genomic DNA from *H. pylori* P12 wild-type *modH5* ON, *modH5* OFF^11^ and the Δ*modH5* deletion mutant^11^ strains (Table 1). The methylated bases, i.e. N^6^-methyladenine (^m6^A), N^4^-methylcytosine (^m4^C), or C^5^-methylcytosine (^m5^C), in the *H. pylori* P12 genome were identified by their IPD ratio; they were then aligned and clustered to identify the motif that constituted the recognition sequence for the active ModH5 methyltransferase in strain P12 (Supplementary Datafile S1). First, the results obtained from the methylome analysis on P12 wild-type (wt) not only agreed with the previously published methylome data for P12^24^, but also revealed an additional novel methylation target sequence, 5′-GA^m6^AGA-3′ whose cognate DNA methyltransferase remained unidentified (Table 1). Second, comparison of the methylome of wild-type P12 (*modH5* ON) to those of *modH5* OFF and Δ*modH5* isogenic mutants indicated that the ModH5 methyltransferase was responsible for the N^6^-adenosine methylation in the sequence 5′-G^m6^ACC-3′.

**Table 1:** SMRT methylome analysis of the *H. pylori* P12 *modH5* ON, *modH5* OFF and Δ*modH5* strains.

Recognition motif^a^	Type	*modH5* ON^b^	Δ*modH5*	*modH5* OFF	Gene^c^	Name
5′-GAAN_8_TAG-3′3′-CTTN_8_ ATC-5′	I	99.7%/99.5%	99.6%/99.6%	99.7%/99.5%	HPP12_0797	Unassigned
5′-GRNAAN_7_TAYC-3′3′-CYNTTN_7_ ATRG-5′	I	96.8%	98.9%	99.5%	HPP12_1508	Unassigned
5′-ATTAAT-3′3′-TAATTA-5′	IIP	98.9%	98.6%	98.4%	HPP12_0488	M.HpyPVII^24^
5′-CATG-3′3′-GTAC-5′	IIP	92.6%	92.6%	92.7%	HPP12_1173	M.HpyPI
5′-GAAGG-3′	IIS	99.9%	99.7%	99.9%	HPP12_0048	M.HpyPV^24^
5′-GAATTC-3′3′-CTTAAG-5′	IIP	98.3%	98.3%	98.0%	HPP12_1389	M.HpyPII^24^
5′-GAGG-3′	IIS	99.6%	99.5%	99.8%	HPP12_0044	M1.HpyAVI^24^
5′-GATC-3′3′-CTAG-5′	IIP	99.9%	99.9%	99.9%	HPP12_0095	M.HpyPIII^24^
5′-GCGC-3′^d ^3′-CGCG-5′	IIP	35.3%	33.7%	34.6%	HPP12_1087	M.HpyPORF1087P (this study)
5′-GTAC-3′3′-CATG-5′	IIP	98.5%	99.6%	100%	HPP12_0510	M.HpyPIV^24^
5′-TCNNGA-3′3′-AGNNCT-5′	IIP	99.7%	99.6%	99.8%	HPP12_1052	M.HpyPVIII^24^
**5**′-GACC-3′	**III**	**99.8%**	**6.7%**	**6.4%**	**HPP12_1497 (this study)**	**M.HpyPIX(this study)**
5′-GAAGA-3′	unknown	30.6%	32.3%	31.7%	unknown	Unassigned
5′-GNGRGA-3′	unknown	99.9%	99.7%	99.9%	unknown	Unassigned

^a^Methylated site underlined in recognition motif. ^b^Percentage of recognition sites detected as methylated in the genome are shown. ^c^Gene assignments as reported by Furuta *et al*.^24^ unless otherwise stated. ^d m5^C modification; signal not strong enough to determine modified base.

Interestingly, our SMRT data indicated that ~6% of 5′-GACC-3′ sites in the *modH5* OFF and the Δ*modH5* deletion mutant strains were methylated (Table 1). A significant proportion of these G^m6^ACC sites (4%) were found to overlap the target sites of other methyltransferases, namely GNGRG^m6^A or TCNNG^m6^A. The remaining ~2% of G^m6^ACC sites in the mutants were called as methylated according to the QV score cut-off of 30, but had significantly low IPD ratios/QV scores compared to scores of other target sites in the same strain (e.g. GNGRGA) and GACC sites in P12 wt (Supplementary Fig. S1). Thus, we do not rule out the possibility that these latter G^m6^ACC sites might be false positives.

### GACC sites are underrepresented in the *H. pylori* genome

**Figure 1:**
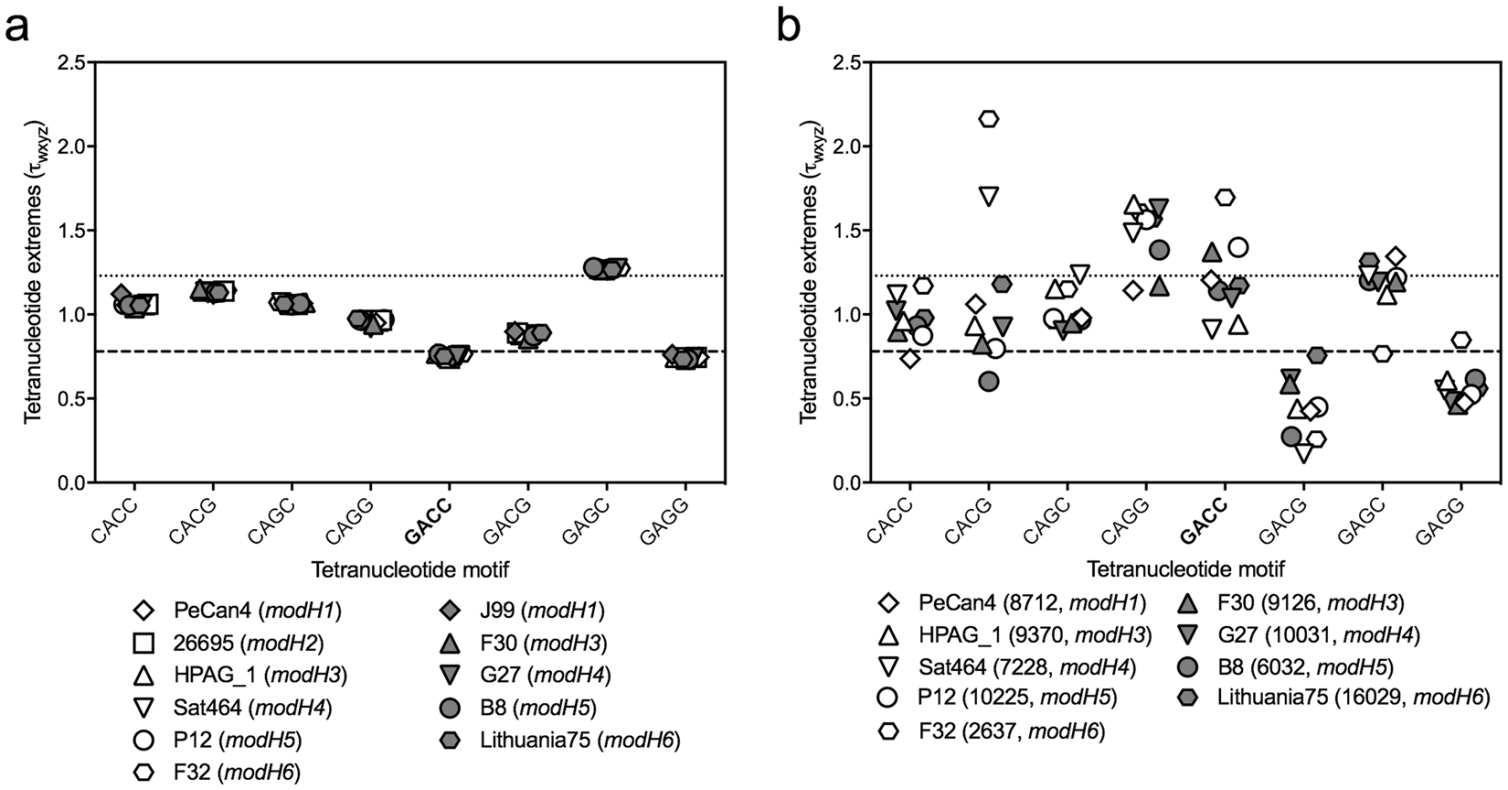
Over- and under-representation of GACC-related tetranucleotide motifs in *Helicobacter* species and naturally competent non-*Helicobacter* species. Tetranucleotide representation in: (**a**) *H. pylori* chromosomal DNA tetranucleotide representation, *modH* type in brackets; and (**b**) *H. pylori* plasmid DNA, plasmid size and *modH* type in brackets. Tetranucleotide extremes were examined using Signature (Institute of Bioinformatics, University of Georgia) to determine Karlin’s tau (τ_wxyz_) values whereby <0.72 (dashed line) or >1.28 (dotted line) indicate significantly underrepresented or overrepresented tetranucleotide motifs, respectively.

The P12 genome contains 2580 GACC sites on the genome and 24 sites on plasmid pHPP12, equating to frequencies of 0.77 sites/kb ssDNA and 1.17 sites/kb ssDNA, respectively. The frequency of GACC sites on the genome was markedly less than the number of sites predicted by the P12 genome GC content of 38.8% (Table 2), indicating a bias against GACC carriage. Identification of tetranucleotide relative abundance extremes, i.e. significant over- or under-representation as described by Karlin *et al*.^28^, among all possible 4-nucleotide sequences within the P12 genome confirmed a significant bias against GACC carriage. The relative abundance (τ_wxyz_-values) of GACC and similar tetranucleotide sequences are shown in Fig. 1. Notably, this bias was conserved in the genomes of all *H. pylori* strains examined regardless of the *modH* type carried (Fig. 1a). In contrast, plasmid DNA from the same selection of *H. pylori* strains did not show a bias against GACC carriage, and instead showed substantial interstrain variation in tetranucleotide skewing. For example, a significant overrepresentation of GACC was observed in the P12 plasmid pHPP12 (Fig. 1b), despite pHPP12 having a lower overall GC content (35%) than the P12 genome. Tetranucleotide composition was also examined in the genomes of various non-pylori *Helicobacter* species but the tetranucleotide skewing was different between each species examined, as well as to that observed in *H. pylori* genomes, and did not include GACC underrepresentation (Supplementary Fig. S2a). In contrast, similar analysis of other naturally competent bacterial species indicated that some other bacterial genera did have conserved tetranucleotide skewing, including *Campylobacter* species also showing a bias against GACC in their genome (Supplementary Fig. S2b). These observations suggest that there is significant selective pressure to restrict the occurrence of GACC in the *H. pylori* chromosome, and that this selection is preserved in *H. pylori* strains independently of whether they carry the *modH5* allele. Moreover, this bias is not observed in other *Helicobacter* species, but can occur in other Epsilonproteobacteria.

**Table 2:** Distribution of GACC sites throughout the P12 chromosome.

**P12 chromosome characteristics** – **genbank accession number CP001217**				
Size	1673813 bp dsDNA/3347626 bp total			
GC content	38.8%			
**Number of GACC sites**	**Forward strand**	**Reverse Strand**	**Total** ^**a**^	
Predicted (based on GC content)	3732	3732	7465	
Predicted frequency (sites/kb)	2.2	2.2	2.2	
Actual	1340	1240	2580	
Frequency (sites/kb)	0.80	0.74	0.77	
**Coding regions**	**Number of genes**	**Number of bases**	**GACC sites**	
			**Number**	**sites/kb** ^**b**^
Open reading frames^c^	1568	1496527	2300	1.54
rRNA	6	8988	50	5.56
tRNA	36	2837	40	14.1
Frameshift/inactive^c,d^	14	28100	44	1.57
Total	1624	1536452	2434	1.58
**Intergenic regions**	na	137361	146	1.06

^a^Calculated using both strands of chromosomal DNA i.e. 3347626 bp. ^b^Calculated using gene length without considering both strands of DNA independently. ^c^Overlapping sequences counted only once in total number of bases; GACC sites within overlapping sequences counted once only. All genes HPP12_0001 to HPP12_1582 considered as coding rather than intergenic for purposes of GACC content analysis. ^d^Inactivated genes are HPP12_0209, 0251, 0314, 0436, 0466, 0467, 0600, 0706, 1319, 1338, 1351, 1378, 1510 and 1512.

### The ModH5 recognition site GACC is overrepresented in motility and outer membrane protein genes

**Figure 2:**
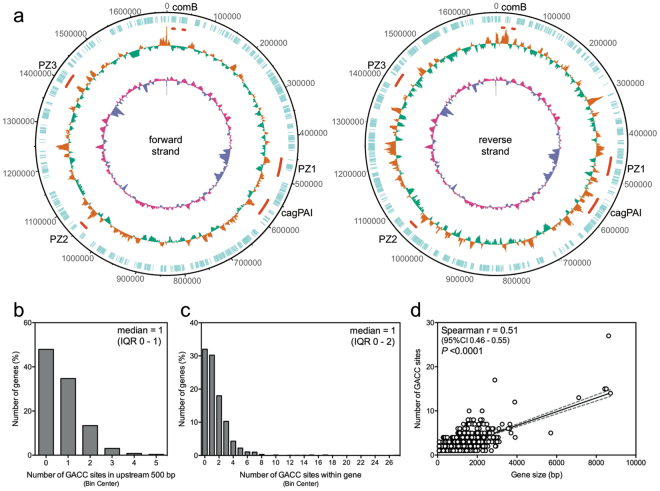
GACC distribution on *H. pylori* P12 genome. (**a**) Forward strand (1340 GACC sites) and reverse strand (1240 GACC sites) of the P12 genome. From outside track: turquoise-ORFs; GACC frequency, orange–high, green–low; %GC content, pink–high, purple–low. Red bars denote areas of interest in the P12 genome (clockwise): comB, including type IV secretion system (TFSS) 2; PZ1–plasticity zone 1, including TFSS4; cagPAI–*cag* PAI pathogenicity island, including TFSS1; PZ2–plasticity zone 2; PZ3–plasticity zone 3, including TFSS3. (**b**) Distribution of the number of GACC sites in 500 bp region upstream of all P12 genes. (**c**) Distribution of the number of GACC sites in all P12 genes. (**d**) There was a significant positive correlation between the number of GACC sites within a gene and the length of the gene (Spearman’s correlation).

The ModH5 GACC target sequence was widely distributed throughout the genome and equivalently present on each strand (Fig. 2a and Table 2). The proportion of GACC sites in intergenic regions (5.7%) was significantly less than expected given that 8.2% of the P12 genome is intergenic (Chi square *P* < 0.0001; odds ratio 0.67, 95% CI 0.57 to 0.79). Close examination of the position of GACC sites with respect to all individual gene loci showed that GACC sites were commonly found both proximal to and within coding regions, with 52% of the annotated P12 genes having one or more GACC site within the 500 bp upstream of the start codon (Fig. 2b), and 68% of genes containing at least one GACC site within the coding region (Fig. 2c).

Although there was a direct correlation between gene size and number of GACC sites within the coding regions (Fig. 2d), a subset of genes showed GACC frequency above that expected from this correlation with gene size. Due to the low prevalence of GACC in the P12 genome, a comparison of individual gene size against its GACC frequency (GACC sites per kb) indicated that genes <500 bp in length had dramatically exaggerated GACC frequency, which is likely a statistical artefact due to the low frequency of GACC throughout the genome (Supplementary Fig. S3a). After excluding this subset of genes (i.e. <500 bp in length), the remaining 902 genes were shown to carry a median of 1.66 GACC sites/kb dsDNA (interquartile range 1.15–2.46) (Supplementary Fig. S3b). Consequently, genes with a GACC frequency higher than the upper interquartile range of 2.46 GACC sites/kb dsDNA (226 genes) were considered to have significant GACC overrepresentation, of which genes with 6 or more GACC sites are listed in Table 3.

**Table 3:**
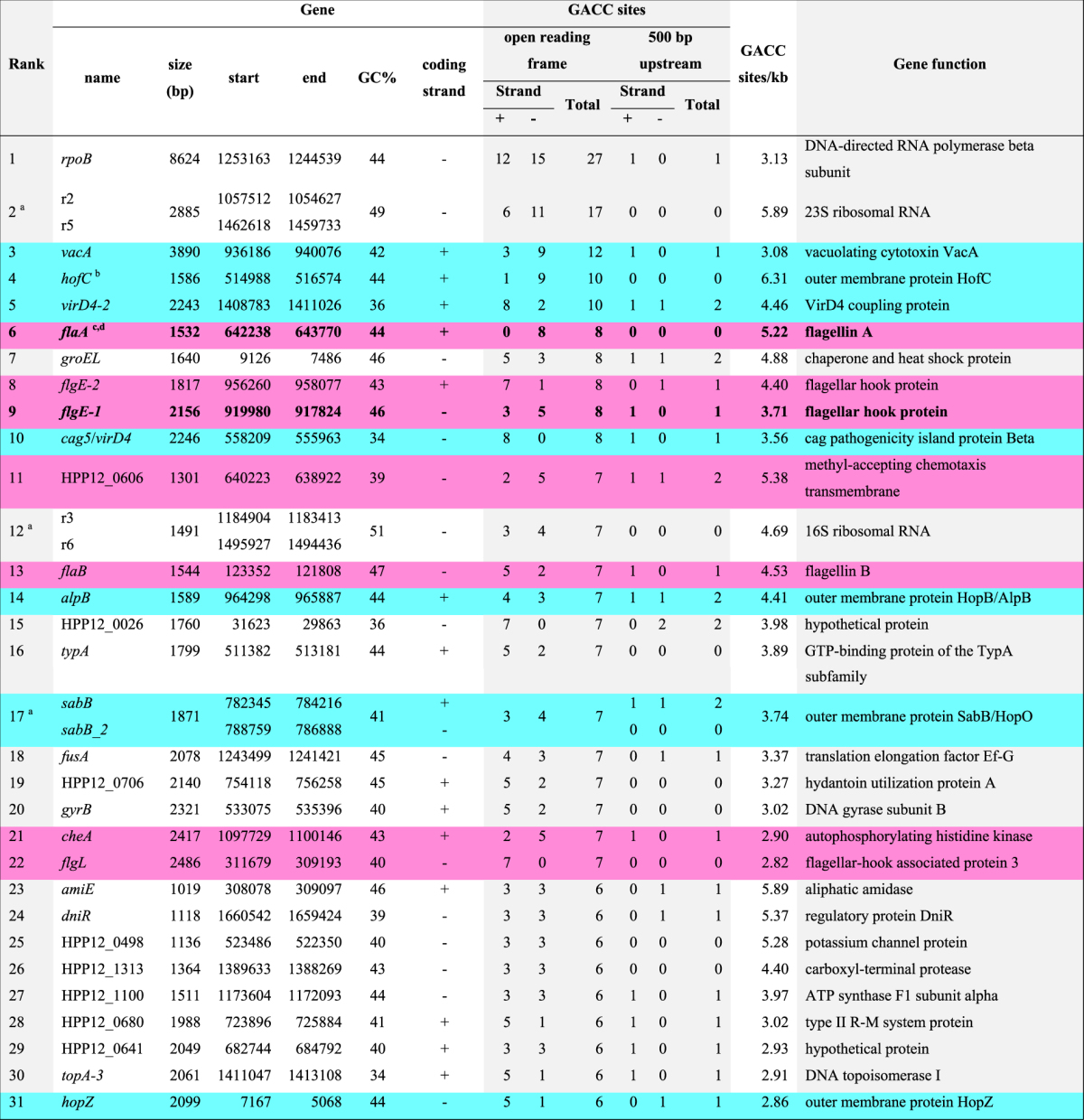
List of the top 31 GACC-hypermethylated genes. ^a^Duplicated ORFs with 100% nucleotide identity share the same rank. ^b^Blue-shaded rows denote outer membrane and virulence proteins. ^c^Pink-shaded rows denote motility-associated genes. ^d^Bolded text denote genes previously identified to be regulated by *modH5* ON/OFF status^11^. Genes are ranked firstly according to the number of GACC sites within each ORF (≥6), and secondly according to the number of GACC sites/kb for each ORF. Only >500 bp genes with >2.46 GACC sites/kb were considered.

Notably predominant among these top 31 genes are two functional groups that are critical for *H. pylori* pathogenesis, namely motility/chemotaxis-associated proteins (seven genes) and adhesion/cytotoxicity proteins (eight genes). Amongst the latter group, the genes encoding HopZ, VacA and Cagβ/VirD4 are of particular interest with respect to *H. pylori* pathogenesis. Of the five adhesin genes, three (*hopZ*, *sabB* and *sabB_2*) have two other known phase variable modes of regulation i.e. translational regulation via dinucleotide repeats in the signal sequence and transcriptional regulation via a homopolymeric T-tract in the promoter region^29,30^.

### *H. pylori* motility is regulated by *modH5* phase switching

**Figure 3:**
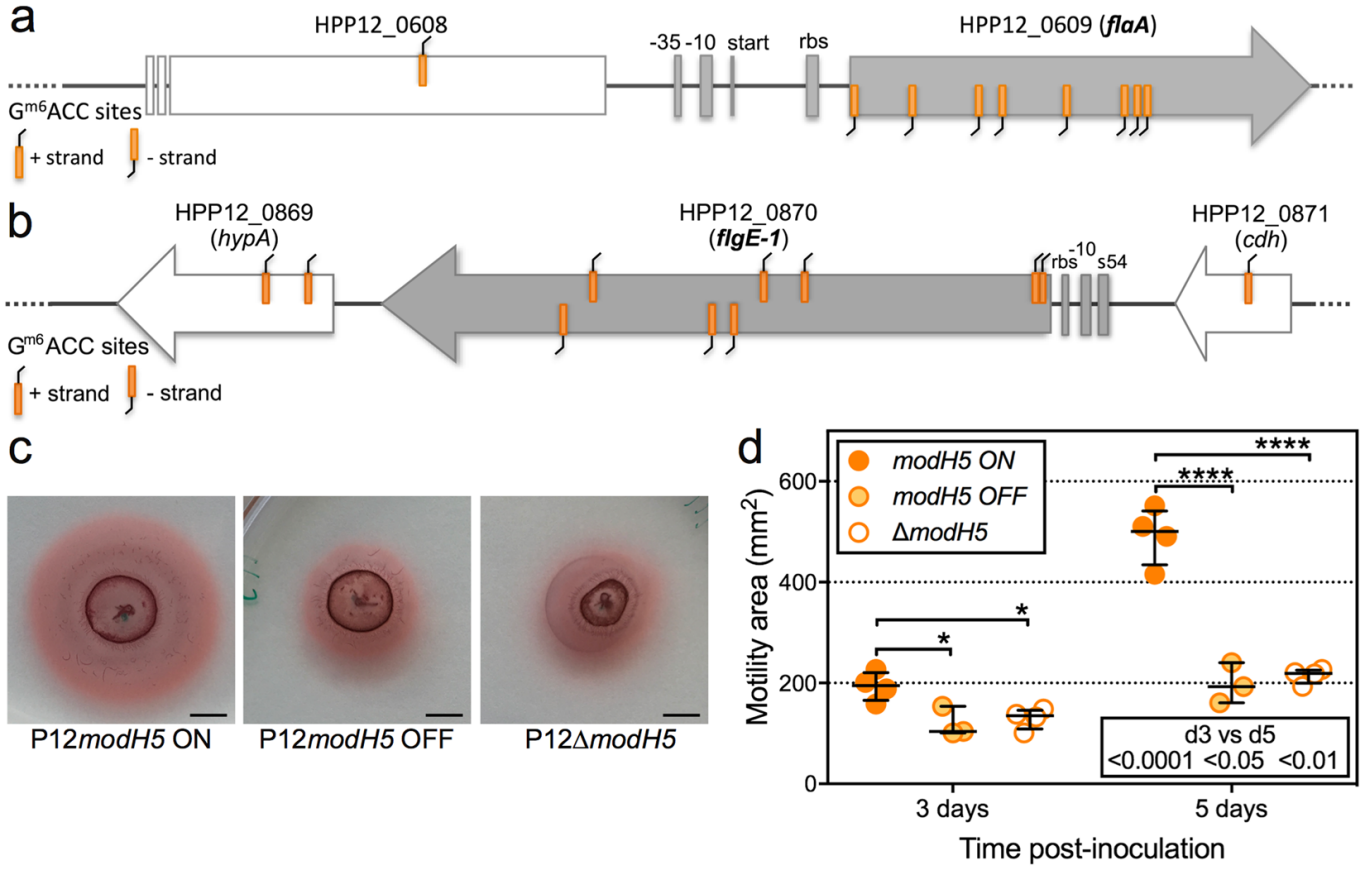
ModH5 ON/OFF state differentially modulates *H. pylori* P12 motility. GACC ModH5-target sites are overrepresented in essential motility genes *flaA* (**a**) and *flgE-1* (**b**), which have both previously been shown to be transcriptionally modulated by *modH5* ON/OFF state. The schematics show the known promoter features (filled boxes) and open reading frames (filled arrow) of the motility genes, and their flanking genes (open arrows). (**c**) Representative motility images of *modH5* ON (P12 wt), *modH5* OFF and Δ*modH5* strains stabbed into soft agar; images captured at 5 days post-inoculation; the addition of tetrazolium chloride to the soft agar allowed visualisation of the distance travelled (outer red ring) over time; scale bar = 5 mm. (**d**) Comparative motility of *modH5* ON (P12 wt), *modH5* OFF and Δ*modH5* strains; bars denote mean ± SD (each symbol represents the mean of technical replicates from an individual experiment; P12 wt and P12Δ*modH5*, N = 4 independent experiments; P12 *modH5* OFF, N = 3 independent experiments); P-values determined by two-way ANOVA, *P < 0.05, ****P < 0.0001 denote significant enhancement in motility of P12 wt compared to isogenic OFF strains; significant migration of each strain between d3 and d5 indicated beneath each d5 dot plot.

Motility is crucial for *H. pylori* colonization of the gastric mucosa^31,32^. We previously reported that four of the five genes identified by transcriptional microarray as members of the ModH5 phasevarion of *H. pylori* strain P12 are motility-associated^11^. These genes were *flaA* (*hpp12_0609*), *flgE-1* (*hpp12_0870*), *fliK* (*hpp12_0904*) and *hpp12_0255*, which respectively encode the major flagellin subunit A, flagella hook protein, flagella hook-length control protein, and a homolog of the *Salmonella* flagella-associated chaperone FliJ that has been shown to be essential for full motility and adhesion in *H. pylori* ^17^. In this present study, we bioinformatically identified nine motility genes as having GACC overrepresentation, of which seven were in genes that carry GACC at very high frequency (Table 3). In particular, *flaA* (Fig. 3a) and *flgE-1* (Fig. 3b) carried a very high frequency of GACC sites compared to the remainder of the P12 genome and were among the top 10 genes showing GACC overrepresentation (Table 3). The distribution of GACC sites in the remaining motility genes included in Table 3 are shown in Supplementary Fig. S4.

Our bioinformatics analysis, together with the previous transcriptional ModH5 phasevarion analysis^11^, suggested that ModH may be a novel regulator of *H. pylori* motility. We therefore hypothesised that although transcription of individual motility genes was only moderately affected by *modH5* ON/OFF state, the combined impact from differential expression of multiple motility-associated genes would have a measurable effect on *H. pylori* motility phenotype(s). To test this hypothesis, we compared the motility of wild-type P12 *modH5* ON strain with that of the P12 *modH5* OFF and P12∆*modH5* strains by stab-inoculating the strains onto motility agar plates (Fig. 3c). P12 wt and the mutant strains showed significant migration through the soft agar by 5 days post-inoculation (dpi) compared to 3 dpi (Fig. 3d) indicating that all three strains were motile. However, motility of P12 wt *modH5* ON was significantly enhanced compared to the mutant strains at both 3 dpi (~50% increase, *P* < 0.05) and 5 dpi (~2-fold increase, *P* < 0.0001) (Fig. 3d). These findings demonstrate that ModH5 plays a key role in modulating *H. pylori* motility.

**Figure 4:**
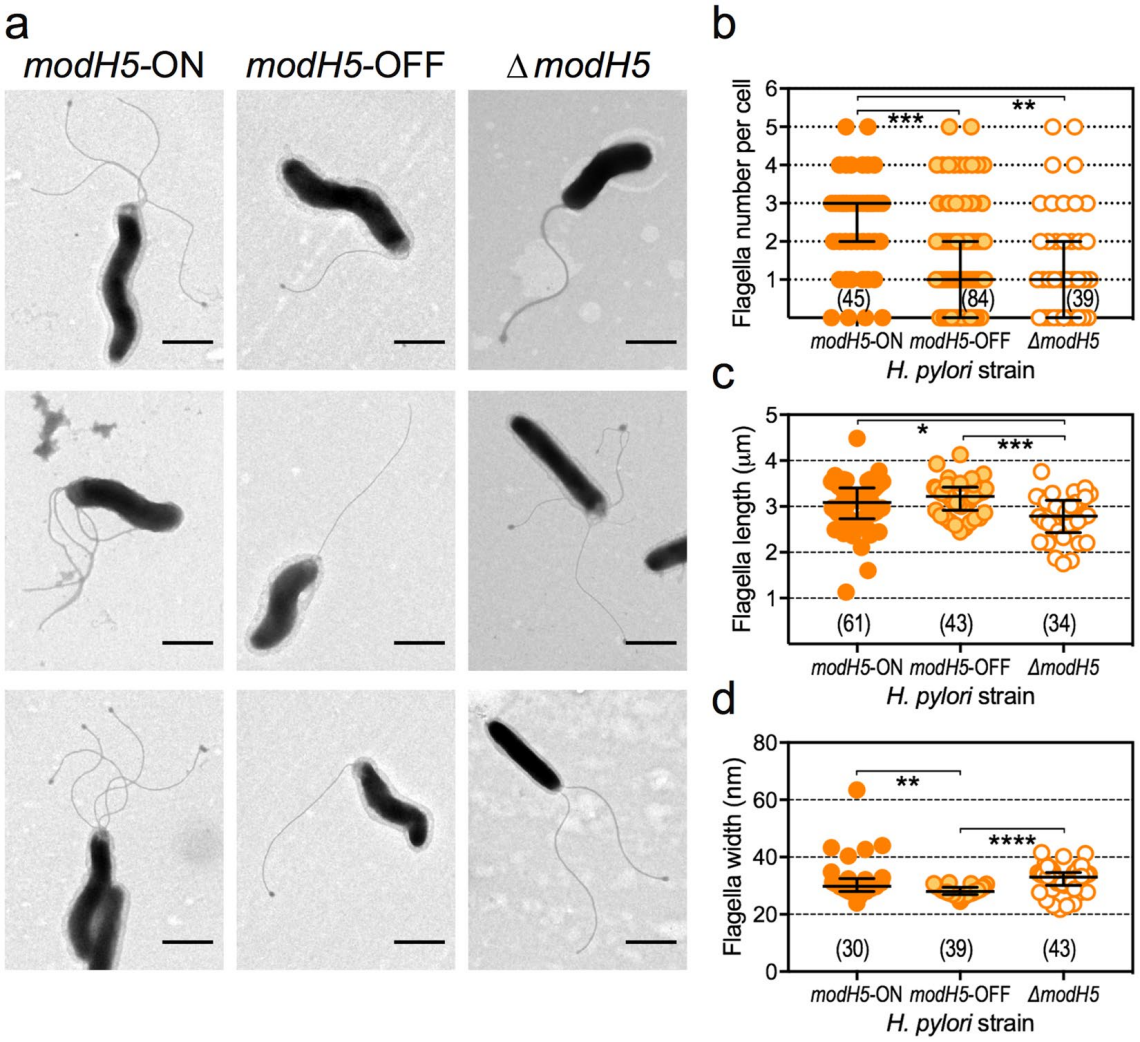
ModH5 ON/OFF state differentially modulates *H. pylori* P12 flagella number. (**a**) Paraformaldehyde-fixed, broth-cultured *H. pylori modH5* ON (P12 wt), *modH5* OFF and Δ*modH5* strains were adsorbed onto formvar copper grids, negative stained with ammonium molybdate and imaged by transmission electron microscopy; 3 representative images shown of each strain (scale bar = 1 μm). Individual bacterial cells were assessed using Fiji ImageJ software for (**b**) number of flagella per cell (magnification 8000x), (**c**) flagella length (magnification 8000x), and (**d**) flagella width (magnification 50,000x). P-values were determined by Kruskal-Wallis test with Dunn’s multiple comparison post-test; *P < 0.05, **P < 0.01, ***P < 0.001, ****P < 0.0001; all other comparisons not significantly different. Sample sizes for each group shown in parentheses.

Given the differential expression of genes encoding the flagella structural components flagellin A (*flaA*) and the hook protein (*flgE-1*), we also visually assessed flagella of the wt and mutant strains by transmission electron microscopy (Supplementary Fig. S5). P12 wt had on average three times as many flagella per bacterial cell than the two mutant strains (Fig. 4a and b), with ~30% of *modH5*-OFF or Δ*modH5* cells being aflagellated (Supplementary Fig. S5d). This substantial difference may be sufficient to account for the observed enhanced motility of the *modH5*-ON strain. We also detected minor differences in the length (Fig. 4c) and width (Fig. 4d) of flagella on the different strains, however these differences did not appear to correlate with *modH5* ON/OFF-status.

### GACC sites flanking the *flaA* promoter are sufficient for ModH5-dependent modulation of promoter function

Both *flaA* and *flgE-1* had G^m6^ACC sites upstream of, and within, the ORF. In particular, the GACC methylation profile of *flaA* included 8 G^m6^ACC sites throughout the *flaA* gene, all on the template strand and starting 14 bp into the ORF, and an additional G^m6^ACC site on the coding strand 507 bp upstream of the *flaA* transcriptional start site (Fig. 3a). The mechanism(s) by which upstream and/or intragenic ModH target motifs contribute to phase variable *H. pylori* epigenetics may provide important insight into *H. pylori* pathogenesis. We were therefore interested in using *flaA* as a model system to identify specific methylation sites involved in ModH5-mediated gene regulation.

**Figure 5:**
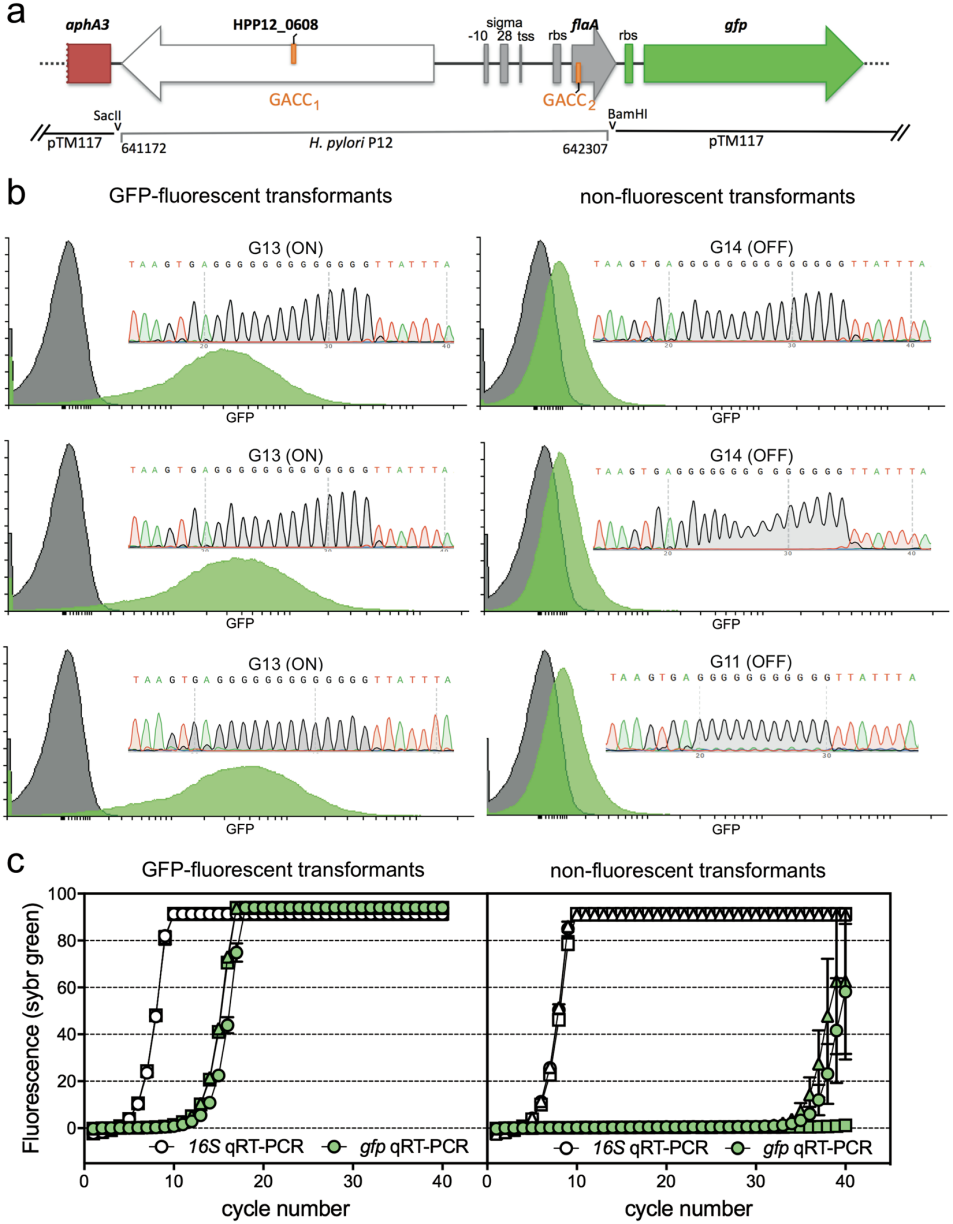
ModH5-mediated methylation of the *flaA* promoter region modulates downstream gene expression. (**a**) pTM117-*flaA* reporter construct design. DNA containing GACC sites upstream of, and at the start of, the P12 *flaA* ORF was inserted upstream of a promoterless *gfpmut3* gene within plasmid pTM117^33^. (**b**) Transformation of *H. pylori modH5* strain 7.13 resulted in both GFP-fluorescent and non-fluorescent kanamycin-resistant transformants as measured by flow cytometric analysis of transformants (green peak) compared to parental 7.13 (grey peak); x-axis denotes GFP fluorescence intensity and y-axis denotes number of cells. Inset chromatograms of the polyG-tract in *modH5* gene of each transformant showing the GFP-fluorescent clones were *modH5*-ON and the non-fluorescent clones were *modH5*-OFF. (**c**) *gfp* mRNA levels in GFP-fluorescent versus non-fluorescent transformants as determined by qPCR using *16S*-specific and *gfp*-specific qPCR of random primed cDNA generated from bacterial RNA. Three independent transformants were assessed per fluorescence phenotype; each symbol type (circle, triangle or square) represents an individual clone; open symbol = *16S* qRT-PCR, filled symbol = *gfp* qRT-PCR; each point represents mean (±SD) of technical triplicates.

We constructed a *flaA* expression reporter plasmid by cloning 1.1 kb of the P12 genome containing the *flaA* promoter into the promoterless green fluorescent protein (GFP)-reporter vector pTM117^33^. The resultant *flaA*-*gfp* transcriptional fusion plasmid is designated pTM117-*flaA*. Previous reporter studies examining stimuli-mediated regulation of the native *flaA* promoter typically included not only the *flaA* upstream sequence, including the transcriptional start/σ28/−10/−35 promoter region (nt 642,138 to 642,188), but also a proportion of the *flaA* coding region in the upstream sequence of the reporter construct to drive reporter gene expression^34–36^. Therefore, taking into account the possibility that elements within the *flaA* coding region might somehow be involved in *flaA* promoter regulation, we included both the GACC located upstream of *flaA* ORF (GACC_1_) and the first internal GACC site adjacent to the start codon (GACC_2_), in the promoter region of pTM117-*flaA* to drive *gfp* expression (Fig. 5a). Transformation of P12 with this construct was repeatedly unsuccessful, which is in contrast to its ready acceptance of linear DNA but is typical of this strain for circular plasmid DNA. Therefore this construct was used to transform *H. pylori* strain 7.13 that also carries the *modH5* allele^11^, but is more readily transformed by plasmid DNA than most *H. pylori* strains. Unlike the P12 *modH5* that contains a short, relatively low-frequency switching *modH5* G_10_-tract, quantitative sequence analysis of the 7.13 *modH5* polyG-tract length using specific fluorescent-tagged primers showed that the parent 7.13 wt strain was a mixed population of *modH5* ON (G_13_) and OFF (G_11_, G_12_ & G_14_) at 43% versus 57%, respectively. This diversity in G-tract length validated the inherent capability of the *modH5* allele in 7.13 to phase vary at high frequency under the experimental conditions used.

Transformation of 7.13wt by plasmid pTM117-*flaA* resulted in a mixture of kanamycin-resistant (Km^R^) transformants that were either GFP-fluorescent or non-fluorescent. Transformants were randomly selected for *gfp* expression analysis by flow cytometry, and sequence analysis across the *modH5* polyG tract (Fig. 5b). This analysis showed a direct correlation between *gfp* expression and ModH5 function in that GFP-fluorescent and non-fluorescent transformants had in-frame and out-of-frame *modH5* G-tracts, respectively. Quantitative analysis of *gfp* mRNA level indicated a dramatic defect in *gfp* transcription by the *modH*-OFF transformants compared that in the *modH*-ON transformants (Fig. 5c). In contrast, 16S rRNA expression was indistinguishable between the different strains. Together these results suggest that *flaA* promoter activity can be modulated by ModH5 phase variation, likely via altered methylation of the two GACC sites in the promoter construct.

### GACC_1_ is essential for ModH5-dependent *flaA* promoter-regulation

**Figure 6:**
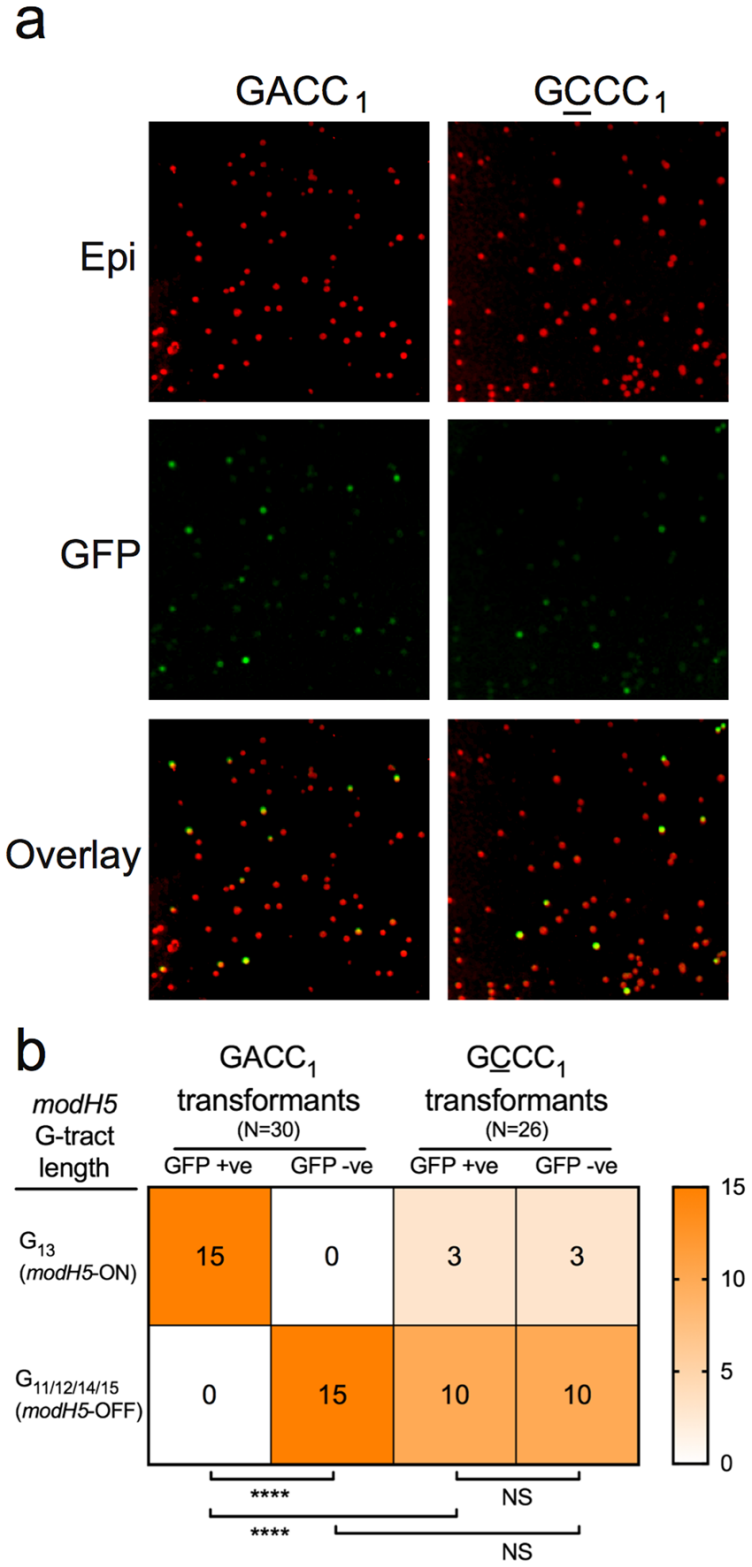
ModH5-mediated methylation of the *flaA* promoter region at site GACC_1_ modulates downstream gene expression. (**a**) pTM117-*flaA* reporter construct carrying either GACC_1_ (wt promoter) or GCCC_1_ (A > C synonymous substitution mutant) was used to transform *H. pylori modH5* strain 7.13 wt; “Epi” - total population of kanamycin-resistant transformants imaged by epi-luminescence (colonies false coloured red in ImageJ); “GFP” - fluorescent transformants imaged using a GFP-specific filter (colonies false coloured green in ImageJ); “Overlay” shows proportion of GFP-fluorescent transformants (yellow/green) to non-fluorescent transformants (red). (**b**) Sequencing of the *modH5* G-tract in GFP-fluorescent (GFP +ve) and non-fluorescent (GFP −ve) transformants carrying pTM117-*flaA* with GACC_1_ versus GCCC_1_ showed that the correlation between ModH5 activity and P12 *flaA* promoter function was uncoupled upon loss of the upstream ModH5 methylation site (GACC_1_). P-values were determined using Fisher’s exact test; ****P < 0.0001, NS = not signficant.

In order to assess the contribution of specific G^m6^ACC sites to *flaA* promoter function, we modified the methylated nucleotide in GACC_1_ of pTM117-*flaA* from A to C (i.e. GCCC, a sequence no longer recognised by ModH5). The resultant construct, designated pTM117-*flaA*-GCCC_1_, was introduced in parallel with the wt promoter construct pTM117-*flaA* into *H. pylori* 7.13 wt. Interestingly, the mutant *flaA* promoter construct produced a mixture of GFP-fluorescent and non-fluorescent transformants in a similar manner to the wt *flaA* promoter construct (Fig. 6a). However, whilst *modH5* G-tract sequencing of 30 randomly selected pTM117-*flaA* transformants revealed a direct correlation between *gfp* expression and *modH5* ON status (P < 0.0001, Fisher’s exact test) (Fig. 6b), sequencing of 26 pTM117-*flaA*-GCCC_1_ transformants indicated that this correlation was lost as a result of GACC_1_ to GCCC_1_ substitution (Fig. 6b). These observations suggest that the upstream ModH5 target sequence plays a direct and pivotal role in epigenetic regulation of *flaA* promoter activity.

## Discussion

*H. pylori* strains possess multiple methyltransferases, including orphan methyltransferases, but the biological role of the majority of these methyltransferases remained unknown^16,37^. We have previously reported that the activity of the *H. pylori* phase variably-regulated DNA methyltransferase ModH5 results in the coordinated regulation of multiple genes. This ModH5-controlled phase variable regulon, or phasevarion, includes various motility-associated genes^11^. These primary findings were recently bolstered by a subsequent characterisation of the *H. pylori* J99 ModH1 phasevarion, which was shown to also include motility-associated genes^19^. Despite this, the mechanism(s) by which random ModH5-phase switching controls gene expression remained uninvestigated. To elucidate the molecular basis of ModH5-associated gene regulation, a crucial prerequisite is to identify the ModH5 methylation target site. In this study, we used whole-genome SMRT sequencing to confirm that ModH5 is a functional N6-methyladenosine methyltransferase, and to identify its methylated target sequence as 5′-G^m6^ACC-3′. We also demonstrated that P12 motility is modulated by ModH5 activity, and that *modH5* ON phase correlates with enhanced P12 *flaA* promoter activity compared to *modH* OFF phase. To our knowledge, this is the first reported phenotypic evidence of *H. pylori* epigenetic motility regulation. These findings, together with previous transcriptional analyses on the ModH5 and ModH1 phasevarions, strongly suggest an important role for ModH phasevarions in virulence gene regulation and *H. pylori* pathogenesis.

The two ModH enzymes characterised to date, ModH5 and ModH1, methylate at target sequences 5′-G^m6^ACC-3′ (this study) and 5′-GWC^m6^AY-3′^24^, respectively. These target sequences are both unique to *H. pylori*. Interestingly, GACC sites are significantly underrepresented in the chromosomal DNA but not in the resident plasmids of various *H. pylori* strains nor the genomes of other *Helicobacter* species. Underrepresentation of restriction-modification target sites has not previously been reported for a type III restriction-modification target sequence, although it is commonly associated with type II restriction-modification systems, particularly those recognising palindromic targets^38^. Moreover, conserved avoidance of a restriction-modification target sequence within a bacterial genome seems to be more common among anciently acquired restriction-modification systems compared to newly acquired systems^39^. The conservation of this bias against GACC in the *H. pylori* genome would therefore suggest a long-term presence of this system in *H. pylori* strains, and is in keeping with the *modH5* allele being one of the most prevalent types among the strains examined previously^11^. Accordingly, we hypothesize that the bias against GACC sites is due to long standing selective pressure that favours strategic positioning of ModH5 target sites throughout the genome, further highlighting a role for ModH5 in *H. pylori* gene regulation.

Epigenetic regulation in a large number of bacteria including *E. coli*, *Salmonella* and *Caulobacter* have been well described and reveal a variety of mechanisms for mediating gene regulation^40^. A recurrent theme among these mechanisms is that DNA methylation can alter interactions between regulatory proteins and DNA-binding sites, directly regulating transcription^41^. In contrast, very little was known about the mechanism of epigenetic gene regulation in *H. pylori*. In this study, the ModH5 methylation motif GACC was found to be significantly underrepresented in the intergenic regions compared to the coding genome of P12, suggesting that upstream GACC sites are likely to be under positive selective pressure. For *flaA*, whose expression is increased in *modH5* ON^11^, one GACC site is located ~495 bp upstream (GACC_1_) and another site 73 bp downstream (GACC_2_) of well-defined essential promoter elements^34^. The results of our reporter assay argue that these two GACC sites flanking the promoter are sufficient for transcriptional regulation by *modH5* ON/OFF phasing, and that the distal GACC_1_ site is required for ModH5-mediated *flaA* promoter regulation. More specfically, our data indicates that *flaA* promoter function becomes uncoupled from ModH5 in the absence of GACC_1_ methylation, and control reverts to ModH5-independent regulators of flagella production. Whilst the precise mechanism by which ModH5 methylation of *flaA* promoter GACC site(s) results in gene activation remains to be elucidated, our analysis of GACC distribution also indicated that some other target genes of ModH5 do not contain GACC sites in close enough proximity to the upstream regulatory region to directly impact upon promoter function. For example, the closest upstream GACC of *fliK* (*hpp12_0904*) is 1141 bp upstream of the ribosome binding site. This argues that the mechanism(s) by which ModH5 regulates gene expression might vary from one gene to another. Meanwhile, the promoter regions of some ModH5 phasevarion genes are either unknown (e.g. *fliK*) or sigma factor 54-driven promoters which have distant enhancer binding sites^42^ (e.g. *flgE*). In these cases, the functional distance of GACC sites from such promoters remains speculative. Future investigation into the molecular basis of gene regulation by ModH5 for the various other target genes is warranted to shed light on the diversity of mechanisms involved.

We have also noted a comparative abundance of GACC sites within the coding regions of numerous virulence genes. A similar phenomenon has been described for ModH1 recognition sites in J99^19^. There is recent evidence of gene regulation mediated by methylation of ORF-encoded target sites by the VchM methyltransferase of *Vibrio cholera* ^43^. Among the large number of VchM-regulated genes, there was a significant correlation between the number of target sites within the coding region of a gene and enhanced expression level of the gene, particularly for genes containing more than 4 target motifs. How these sites that are located within coding regions influence gene expression is as yet unknown. However it is well reported that eukaryotic DNA methylation within an open reading frame can influence splicing of the resultant mRNA, thereby providing evidence that DNA methylation may influence not only interactions between DNA and gene regulatory proteins, but also RNA synthesis and/or post-transcriptional processing events^44^.

Flagellar motility is essential for gastric colonization and sub-organ localization within the stomach^45^. Motility consumes vast amounts of energy and therefore needs to be tightly regulated transcriptionally. The mechanisms involved in motility regulation are highly complex and not fully understood. The *H. pylori* flagellum is comprised of three main structural components; the filament composed of flagellins FlaA (the major component) and FlaB (a minor component), the hook-associated proteins FlgK and FlgL, and the hook protein FlgE. Our observation in this study that wild-type P12 (ON) has enhanced motility compared to the P12 *modH5* OFF strain is in line with our previous findings that motility-essential genes *flaA* and *flgE* are increased in expression when *modH5* is ON^11^, highlighting the novel role of ModH5 in motility regulation. However, how does the role of ModH5 in motility regulation reconcile with the previous finding that phase variable regulation of motility is mediated in some strains by slippage of a homopolymeric C_8_-tract within the motility-associated gene *fliP* ^46^? Interestingly, *fliP* expression in *H. pylori* P12 and J99 is fixed “ON” via the alternate CCCCACCC sequence. It is thus possible that phase variable regulation of motility in strain P12 occurs not via *fliP* phase switching but rather through ModH5 phase variable epigenetic regulation of *flaA* promoter activity; this might hold true also for other strains such as J99. Apart from directly regulating *flaA* promoter activity, we postulate that ModH5 could also modulate *flaA* expression indirectly. Expression of *flaA,* which is driven from a sigma factor 28 -controlled promoter, is dependent on environmental signals and also on regulatory systems that ensure the sigma factor 54 -controlled flagella hook is assembled in preparation for the hook-filament transition^47,48^. One of the important regulators of *flaA*, FliK, helps to release sigma factor 28 from the anti-sigma factor 28 factor FlgM in response to environmental cues, thereby making sigma factor 28 factor available for *flaA* expression^49^. Notably, *fliK*, like *flaA* and *flgE*, is also downregulated in the absence of ModH5-mediated methylation^11^, suggesting that ModH5 might also be able to modulate *flaA* expression indirectly through regulation of *fliK* expression. However, given that reduced *fliK* expression typically results in *flgE* over-expression^50^, we hypothesize that phase variable epigenetic modulation of *flaA, flgE* and *fliK* expression might act as an additional ‘rheostat’ to fine tune positively and/or negatively the expression of various motility genes and hence their roles in *H. pylori* colonization, nutrient acquisition and host adaptation.

The recent ‘epigenetics-driven adaptive evolution’ hypothesis suggests that diverse methylomes rather than diverse genome sequences are ideal targets for natural selection^51^, and the inherent genetic mobility of *H. pylori mod* DNA recognition domains provides a novel mechanism for rapid diversification^52^. In line with these notions, we propose that a combination of ModH ON-OFF phase switching, variable DNA-methylation specificity and differing phasevarion composition would generate tremendous diversity crucial for *H. pylori* to adapt to the highly variable and complex host microenvironment whilst evading host immune defense.

Taken together, the findings of this and other recent studies on *H. pylori* methylomes highlight the emerging importance of DNA methyltransferases as a important epigenetic regulator of virulence gene expression and a critical driver of bacterial evolution and adaptation. This study has provided an important basis for further investigation into the underlying molecular mechanisms, knowledge of which is likely to revolutionize our understanding of bacterial epigenetics and its role in *H. pylori* pathogenesis.

## Methods

### Strains and growth culture conditions

*H. pylori* strains were routinely grown from glycerol stocks for 2 days on GC agar (Oxoid, Basingstoke, UK) plates supplemented with 10% (v/v) horse serum (Invitrogen Corp, Carlsbad, CA), vitamin mix and antibiotics (nystatin, 20 μg/ml; trimethoprim, 2.5 μg/ml; vancomycin, 10 μg/ml) in a microaerobic atmosphere as described previously^11^. Plates for cultivation of mutant strains were further supplemented with chloramphenicol (4 μg/ml for routine culture, 10 μg/ml for selection of transformants). *H. pylori* strains used in this study were P12 wild-type (also designated *modH5 ON*; in-frame G_10_ tract; phenotype *modH* ON), P12∆*modH*::*cat* (also designated ∆*modH5*; replacement of *modH* DRD with *cat* cassette; phenotype *modH* OFF)^11^, P12 *modH*OFF::*cat* (also designated *modH5 OFF*; in-frame G_10_-tract substituted to out-of-frame G_6_AG_4_, and region after premature stop codon replaced with a *cat* cassette; phenotype *modH* OFF)^11^, and 7.13 wild-type showing heterogeneous *modH* G-tract lengths.

### Analysis of *modH5* repeat tract

The length of the poly-G repeat tract was determined by Sanger sequencing, using primer pair ModHF_repeat (5′-ATGCCGTGTTAGAGAGTAATAAGAGCGA-3′) and ModHR_repeat (5′-TCTAACTGGACGAGAATGAAGCG-3′) to amplify the repeat regions of *modH5*. 6-carboxyfluoroscein-tagged ModHF_repeat primer was used together with unmodified ModHR_repeat to quantitate proportions of different poly G-tract lengths in parental *H. pylori* strains.

### SMRT sequencing

Genomic DNA was extracted from plate-cultured *H. pylori* strains using the QIAGEN DNeasy blood and tissue genomic DNA kit as per the manufacturer’s instructions. SMRTbell libraries were prepared as previously described^53^ according to the manufacturer’s instructions (PacBio, CA, USA). Briefly, genomic DNA was sheared to an average length of approximately 10 kb using g-TUBEs (Covaris; Woburn, MA, USA), treated with DNA damage repair mix, end-repaired and ligated to hairpin adapters. Incompletely formed SMRTbell templates were digested using Exonuclease III (NEB) and Exonuclease VII (Affymetrix; Cleveland, OH, USA). Sequencing was carried out on the PacBio RS II (Menlo Park, CA, USA) using standard protocols for long-insert libraries.

### Bioinformatic and statistical analysis

Reads were mapped against the P12 genome and plasmid sequences (accession numbers CP001217 and CP001218, respectively). The ModH5 methylation recognition site was identified using the Pacific Biosciences’ SMRTPortal analysis platform (v. 1.3.1) as described previously^54^, and its locations relative to genome features analysed using Artemis^55^ and in-house scripts written in Perl and Python. Circular genome figures were created using DNAPlotter^56^ using data derived from the Prokka annotation and SMRT methylome. Comparative analysis of tetranucleotide extremes in *H. pylori* genomes was performed using the Signature server (Institute of Bioinformatics, University of Georgia; http://www.cmbl.uga.edu/software/signature.html) to determine Karlin’s tau (τ_wxyz_) values whereby values less than 0.72 or greater than 1.28 indicate significantly underrepresented or overrepresented tetranucleotide motifs, respectively. Statisitcal analysis of GACC prevalence and motility was performed using GraphPad Prism software (v6.0 h).

### Motility assay

Approximately 4 × 10^6^ CFU of broth-grown *H. pylori* P12 wild-type or isogenic *modH5* mutant strains was stabbed in 5 µl volumes into triplicate soft agar motility plates (20 ml agar dispensed per plate; Brucella broth (BD Biosciences), 7% (v/v) fetal bovine serum (Gibco), 0.4% (w/v) agar (No. 1, Oxoid) and 40 μg/ml metabolic activity indicator triphenyl tetrazolium chloride (Sigma, UK)). Plates were incubated at 37 °C under microaerobic conditions and bacterial motility was assessed by measuring two perpendicular diameters of metabolically active bacteria across each stab after 3 and 5 days growth. For each experiment, averaged diameters of each strain were converted to area (π(d/2)^2^) of bacterial migration, and data from independent experiments were combined for statistical analysis.

### Transmission electron microscopy

For transmission electron microscopy analysis, 1 mL of cells from Brucella broth-grown wild type and mutant strains was pelleted by centrifugation (3 mins at 8,000 rpm), and resuspended in 1% (w/v) neutral buffered paraformaldehyde (30 mins, room temperature). Fixed cells were washed twice with PBS (pH 7.2) and adsorbed onto Formvar, carbon-coated Cu grids (10 μl per grid). Grids were negatively stained with 0.3% (w/v) ammonium molybdate (15 seconds), air dried, and desiccated until imaging under a Phillips CM120 electron microscope at 80 kV.

### Creation of *flaA*-*gfp* promoter fusion plasmid constructs

The transcriptional fusion of *flaA* (HPP12_0609) to the promoterless *gfpmut3* gene in pTM117 (accession number EF540942)^33^ was constructed by amplification of the promoter region of the *flaA* gene from P12 genomic DNA using primers FlaA_SacII_F (5′-TCCccgcggGAGCTAAATGCTTGGATATATCCAGCAAT-3′) and FlaA_BamHI_R (5′-CGCggatccCATTTTGAGTGAGTGCGGATTGC-3′) to generate a 1136-bp amplicon. This product was cloned into pGEM-T Easy to generate pGEM*flaA*, and confirmed by sequencing. The *flaA* fragment was excised by SacII/BamHI digestion and cloned into the same sites of the transcriptional fusion vector pTM117 to create pTM117-*flaA*. The pTM117-*flaA* plasmid was moved into *H. pylori* strain 7.13 wild-type by natural transformation, and transformants were selected on GC plates containing 10 μg/ml kanamycin. Transformants were graded as GFP-fluorescent or non-fluorescent by their fluorescent intensity detected using LAS-3000 Intelligent Darkbox (FujiFilm) in comparison to *H. pylori* 7.13 wild-type. Sequence integrity was confirmed by Sanger sequencing for pTM117-*flaA* plasmid recovered from three GFP-fluorescent and three non-fluorescent transformants; Southern hybridisation analysis of whole genomic DNA confirmed that the *flaA*-*gfpmut3* fusion was retained in the plasmid and had not integrated into the chromosome of 7.13 transformants.

### GFP expression reporter assays

GFP expression level in *H. pylori* 7.13(pTM117-*flaA*) GFP-fluorescent and non-fluorescent transformants was measured by flow cytometry. Strains were grown overnight in liquid culture; 1.5 ml of each culture was pelleted resuspended in 1 ml 4% (w/v) paraformaldehyde in phosphate-buffered saline (PBS) for 20 mins, then resuspended in 1 ml PBS and passed through a 35 μm cell strainer to remove any bacterial clumps or debris. The samples were then analyzed using a BD LSR-II flow cytometer to collect 100,000 events. Flow cytometry data was analyzed using Flowing software (Cell Imaging Core, Turku Institute for Biotechnology, Finland).

### Quantitative RT-PCR

Overnight Brain Heart Infusion broth cultures (10 ml) of GFP-fluorescent and non-fluorescent 7.13(pTM117-*flaA*) transformants were pelleted, resuspended in 100 μl TE buffer (10 mM Tris/1 mM EDTA [pH 8]) supplemented with 1 mg/ml lysozyme, incubated for 20 mins, 37 °C, before adding 350 μl RLY cell lysis buffer (ISOLATE II RNA mini kit, Bioline) supplemented with 10 mM dithiothreitol, and continuing RNA purification according to the kit protocol for “purifying total RNA from cultured cells and tissue”. RNA concentrations were estimated by Nanodrop (Thermo Fisher) and 500 ng RNA was used for cDNA production using the Superscript III first strand synthesis system with random primers (Thermo Fisher) according to the kit protocol. *gfp* mRNA levels were assessed by qPCR using FastStart Universal SYBR Green Master mix (Roche) with gene-specific primers for *gfp* (gfp_qPCR_F 5′-TGTTCCATGGCCAACACTTG-3′ and gfp_qPCR_R 5′-GCACGTGTCTTGTAGTTCCC-3′) and *16 S rRNA* (Hp547f 5′-CTTAACCATAGAACTGCATTTGAAACTAC-3′ and Hp665r 5′-GGTCGCCTTCGCAATGAGTA-3′^57^). Quantitative PCRs were prepared in triplicate and individual 25 μl reactions contained 0.4 mM primer pair and 2 μl cDNA (≈50 ng RNA) or nuclease-free H_2_O (no-template control). PCR cycling conditions were: 1 cycle (95 °C for 10 min); 40 cycles (95 °C for 15 s, 60 °C for 60 s); melting curve from 65 °C–95 °C, 5 s per 1 °C. Output qPCR data was assembled into amplicon groups and analysed using LinRegPCR (software version 2017).

## Electronic supplementary material

Supplementary Figures

Supplementary Datafile S1
