# Supplementary material for: Methylomic and phenotypic analysis of the ModH5 phasevarion of Helicobacter pylori
Source: Sci Rep. 2017 Nov 23;7:16140. doi: 10.1038/s41598-017-15721-x (PMC5700931; doi:10.1038/s41598-017-15721-x)
Supplement: Supplementary file 1 — Supplementary Figures [file 41598_2017_15721_MOESM1_ESM.pdf]

Methylomic and phenotypic analysis of the ModH5 phasevarion of *Helicobacter pylori*

Yogitha N Srikhanta, Rebecca J Gorrell, Peter M Power, Kirill Tsyganov, Matthew Boitano, Tyson A Clark, Jonas Korlach, Elizabeth L Hartland, Michael P Jennings and Terry Kwok

---

SUPPLEMENTARY FIGURES

Supplementary Figure 1 ModH5 mediates specific methylation of G^{m6}ACC in *H. pylori* P12. ……………………………………………………………………...2

Supplementary Figure 2 Over- and under-representation of GACC-related tetranucleotide motifs in non-pylori *Helicobacter* species and naturally competent non-*Helicobacter* species. …………………………………….....3

Supplementary Figure 3 The GACC frequency (sites/kb) in individual genes is artificially inflated in ≤500 bp genes. 4

Supplementary Figure 4 Location of GACC sites in hyper-methylated motility-associated genes...………………………………………………………...5

Supplementary Figure 5 Full-size transmission electron micrographs images of P12 wt and isogenic mutant strains. 6

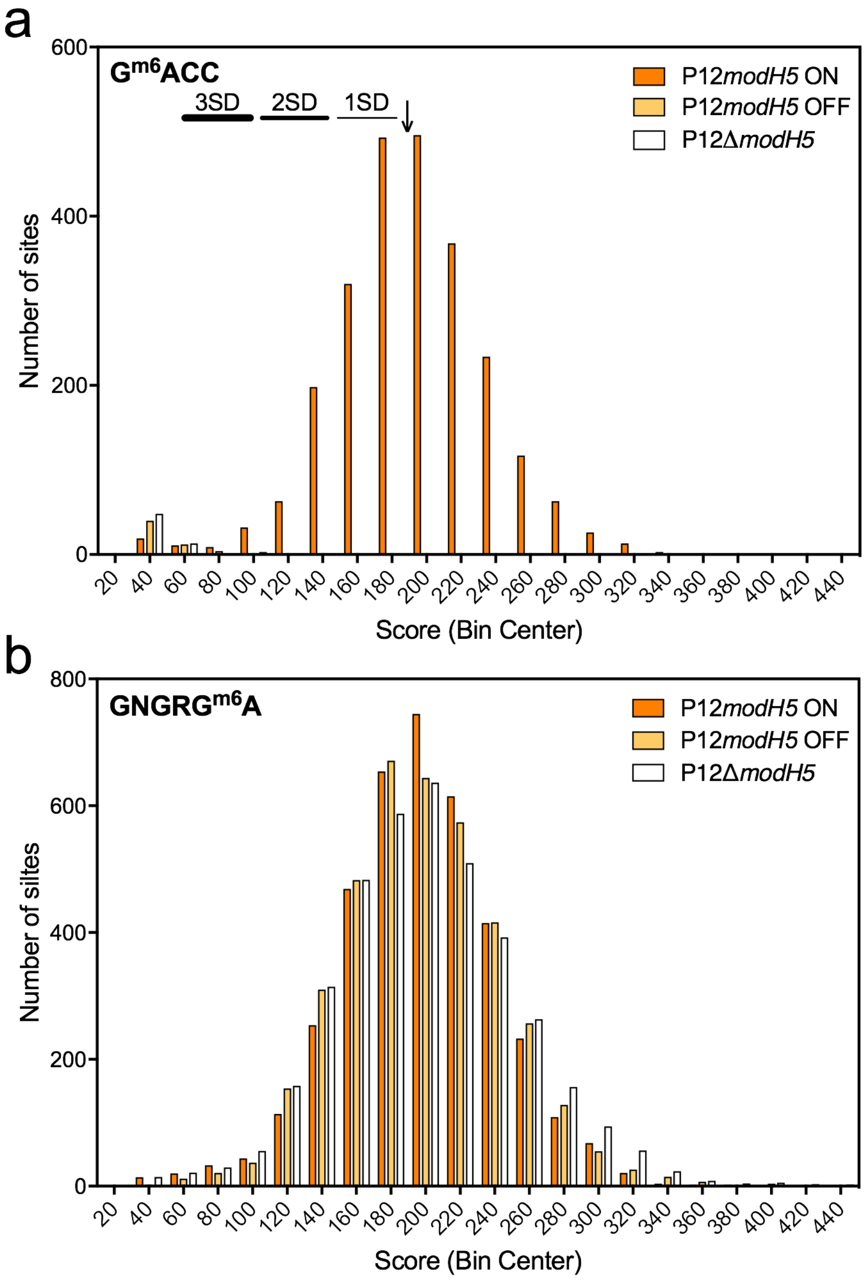

Supplementary Figure 1 ModH5 mediates specific methylation of G^{m6}ACC in *H. pylori* P12.

The ^{m6}A in DNA motif G^{m6}ACC is methylated when the *modH5* polyG tract is in-frame, but not when it is out-of-frame or when *modH5* is partially deleted. (a) Methylated GACC motifs detected in isogenic mutant strains with inactive ModH5 either overlapped sequences recognized by other methyltransferases, or showed very low scores that sat around the cut-off of 30 and were > 2 standard deviations below the mean score for G^{m6}ACC sites found in P12 wild-type carrying a functional ModH5 methyltransferase. (b) No such effect was observed with any other recognition site, e.g. GNGRGA.

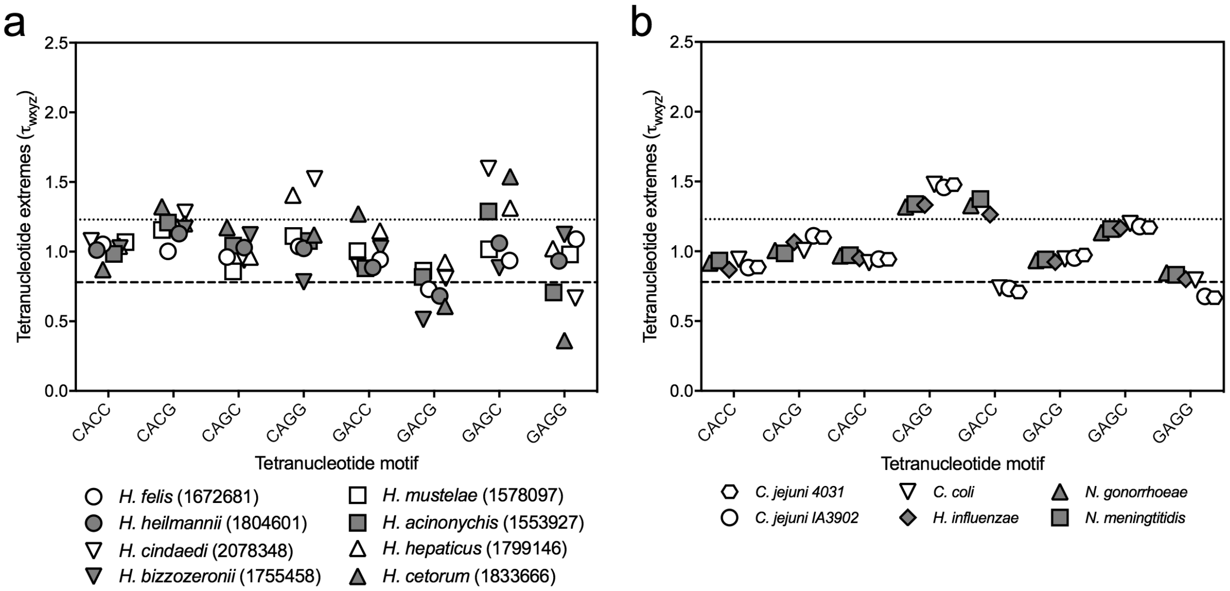

Supplementary Figure 2 Over- and under-representation of GACC-related tetranucleotide motifs in non-pylori *Helicobacter* species and naturally competent non-*Helicobacter* species.

Tetranucleotide representation in: (a) non-pylori *Helicobacter* species, genome size in brackets; and (b) non-*Helicobacter* naturally competent bacterial species. Tetranucleotide extremes were examined using Signature (Institute of Bioinformatics, University of Georgia) to determine Karlin's tau (τ_{wxyz}) values whereby <0.72 (dashed line) or >1.28 (dotted line) indicate significantly underrepresented or overrepresented tetranucleotide motifs, respectively.

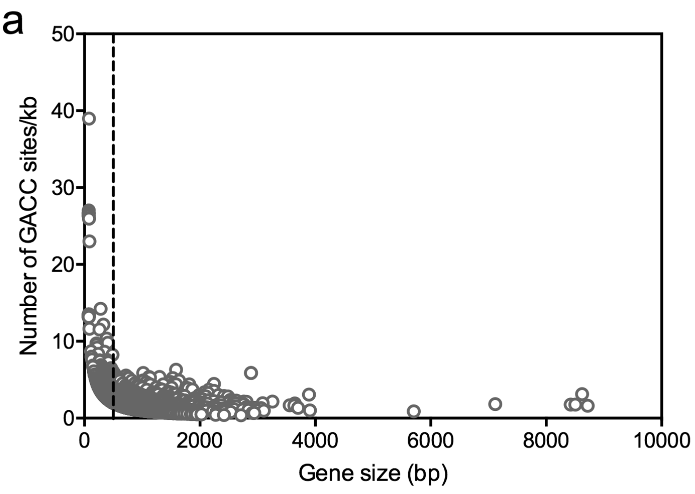

Supplementary Figure 3 The GACC frequency (sites/kb) in individual genes is artificially inflated in ≤500 bp genes.

(a) Plot of the number of GACC sites/kb for individual genes against its own size indicated the smaller genes. This analysis suggested that the GACC frequencies in genes of length ≤500 bp were exaggerated; broken line denotes 500 bp cut-off. (b) Comparison of the GACC frequency in genes of length ≤500 bp (202 genes) versus >500 bp (902 genes) showed the calculation of GACC sites/kb for the smaller genes was significantly elevated. Genes not containing any GACC sites were omitted from this analysis. Median ± interquartile range shown; each symbol denotes an individual gene.

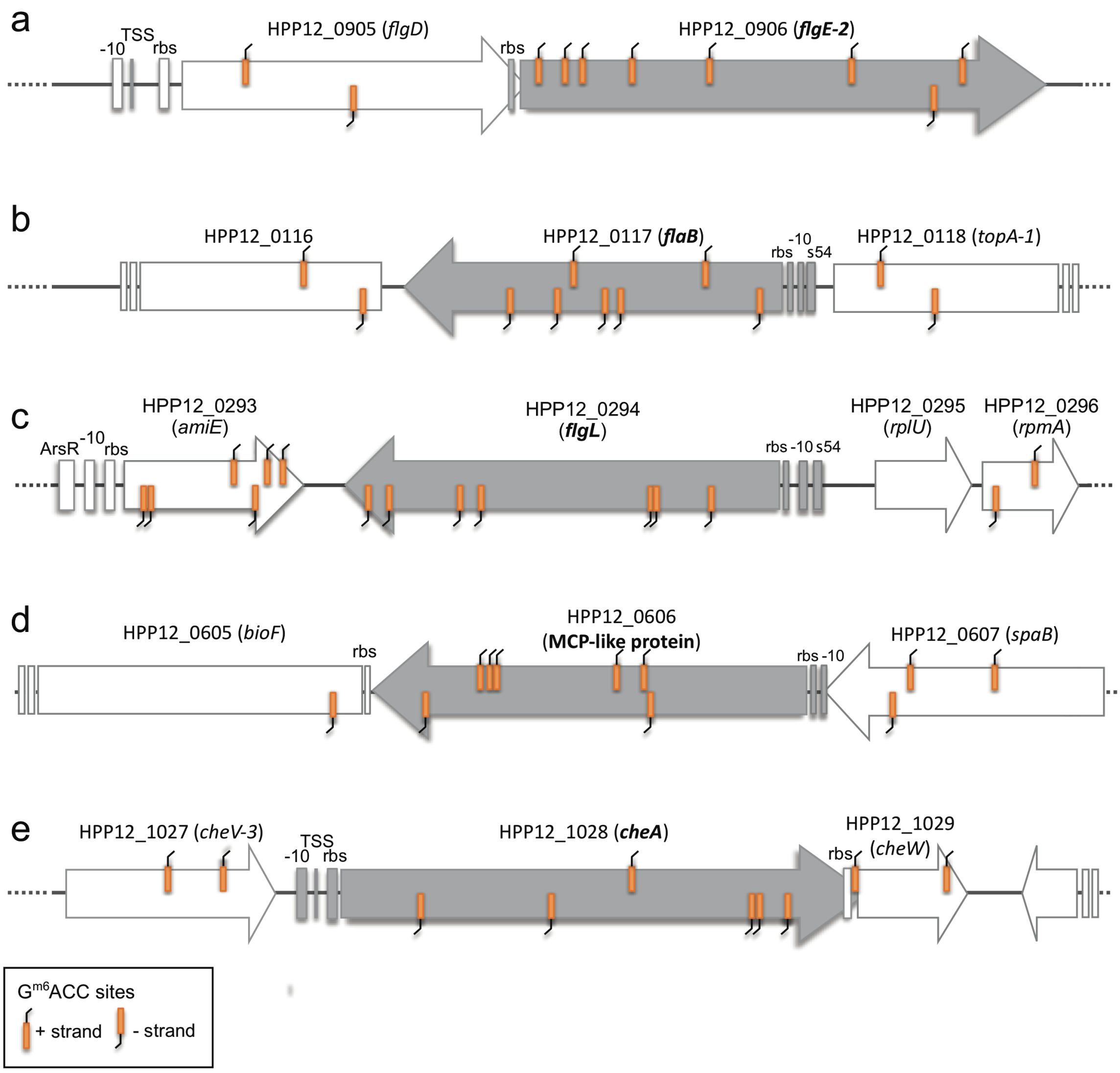

Supplementary Figure 4 Location of GACC sites in hyper-methylated motility-associated genes.

Schematic diagrams of open reading frames (filled arrow) and known promoter features (filled boxes) of motility or chemotaxis associated protein-encoding genes showing significant GACC-overrepresentation: (a) *flgE-2* (flagellar hook protein), (b) *flaB* (flagellin B), (c) *flgL* (flagellar hook associated protein), (d) HPP12_0606 (methyl-accepting chemotaxis protein-like), and (e) *cheA* (autophosphorylating histidine kinase). GACC position and strand indicated by orange boxes as per figure legend; GACC sites in 1kb upstream and downstream regions of each gene are also shown; open arrows indicate flanking genes; next GACC after *flgE-2* ORF (a) is more than 2kb downstream. rbs – ribosome binding site, -10 – Pribnow box, TSS – transcriptional start site, s54 –σ54 binding site, ArsR – ArsR binding site.

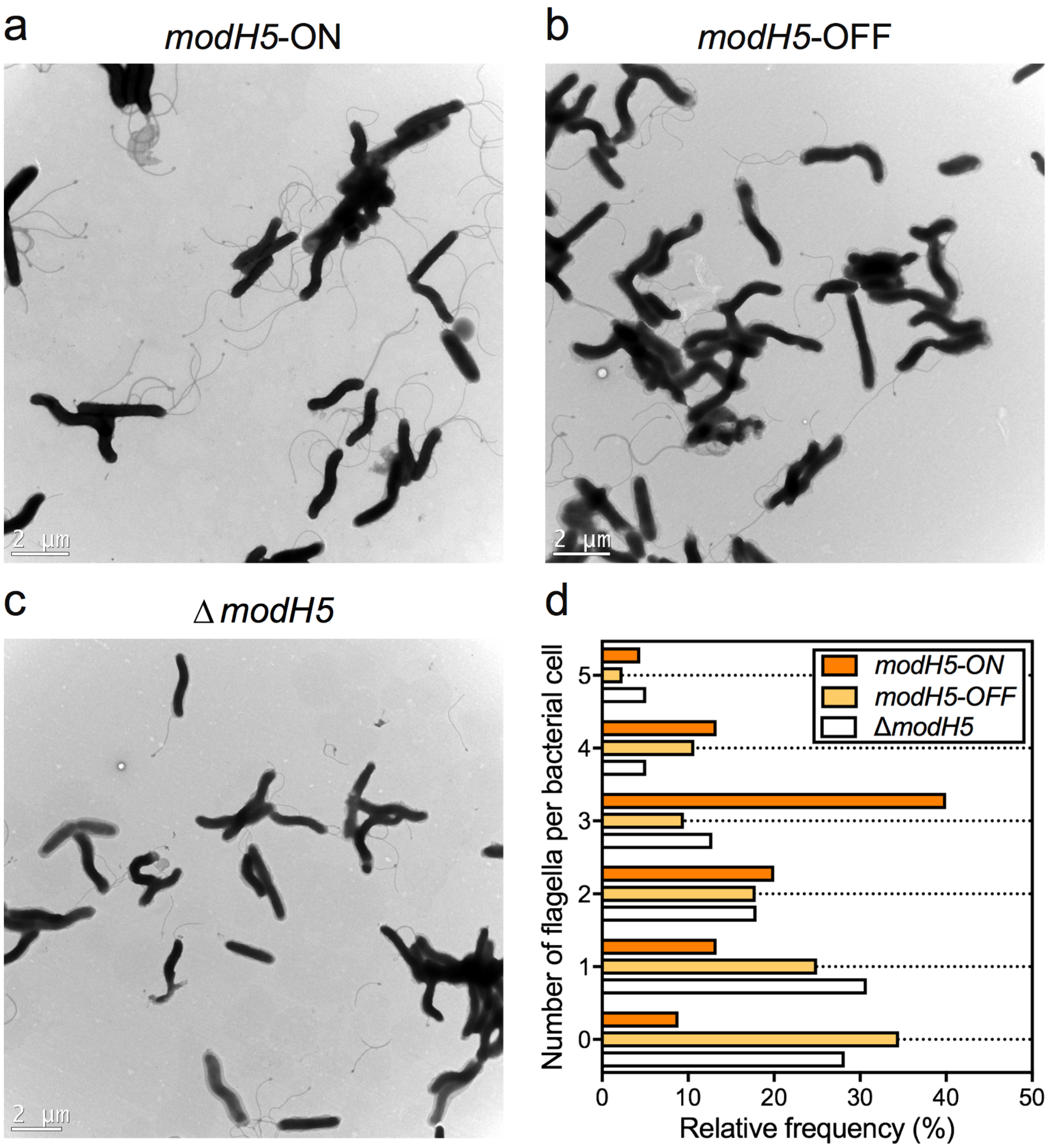

Supplementary Figure 5 Representative full-size transmission electron micrographs of P12 wt and isogenic mutant strains.

Flagella was highly abundant on cells from (a) P12 *modH5*-ON (wt), but were comparatively scare on cells from (b) P12 *modH5*-OFF and (c) P12Δ*modH5*; scale bar = 2 µm (bottom left corner) (d) Relative frequencies of flagella number for each isogenic strain.
